# PET/CT versus conventional CT for detection of lymph node metastases in patients with locally advanced bladder cancer

**DOI:** 10.1186/s12894-015-0080-z

**Published:** 2015-08-21

**Authors:** Firas Aljabery, Gunnar Lindblom, Susann Skoog, Ivan Shabo, Hans Olsson, Johan Rosell, Staffan Jahnson

**Affiliations:** Department of Urology, Linköping University Hospital, Linköping, Sweden; Department of Radiology, Linköping University Hospital, Linköping, Sweden; Department of Clinical and Experimental Medicine, Faculty of Health Sciences, Linköping University, Linköping, Sweden; Regional Cancer Center Southeast Sweden, County Council of Östergötland, Linköping, Sweden

## Abstract

**Background:**

We studied patients treated with radical cystectomy for locally advanced bladder cancer to compare the results of both preoperative positron emission tomography/computed tomography (PET/CT) and conventional CT with the findings of postoperative histopathological evaluation of lymph nodes.

**Methods:**

Patients who had bladder cancer and were candidates for cystectomy underwent preoperative PET/CT using 18-fluorodeoxyglucose (FDG) and conventional CT. The results regarding lymph node involvement were independently evaluated by two experienced radiologists and were subsequently compared with histopathology results, the latter of which were reassessed by an experienced uropathologist (HO).

**Results:**

There were 54 evaluable patients (mean age 68 years, 47 [85 %] males and 7 [15 %] females) with pT and pN status as follows: < pT2-14 (26 %), pT2-10 (18 %), and > pT2-30 (56 %); pN0 37 (69 %) and pN+ 17 (31 %). PET/CT showed positive lymph nodes in 12 patients (22 %), and 7 of those cases were confirmed by histopathology; the corresponding results for conventional CT were 11 (20 %) and 7 patients (13 %), respectively. PET/CT had 41 % sensitivity, 86 % specificity, 58 % PPV, and 76 % NPV, whereas the corresponding figures for conventional CT were 41 %, 89 %, 64 %, and 77 %. Additional analyses of the right and left side of the body or in specified anatomical regions gave similar results.

**Conclusions:**

In this study, PET/CT and conventional CT had similar low sensitivity in detecting and localizing regional lymph node metastasis in bladder cancer.

## Background

Accurate clinical staging of localized or regionally advanced urinary bladder cancer remains a challenge. Lymph node (LN) metastasis is correlated with decreased overall survival (OS), and it is plausible that survival in patients with or without such metastasis can be improved by LN dissection [1–5]. Also, patients with LN involvement may be candidates for preoperative chemotherapy if considered for cystectomy [6–10]. Preoperative staging of regional LNs has relied on radiological imaging to identify nodes that meet anatomical criteria. Conventional computed tomography (CT) and magnetic resonance imaging (MRI) are frequently employed as diagnostic tools for staging in evaluation of muscle-invasive bladder cancer. Both these methods use LN size as a criterion for diagnosis and false-negative rates can be as high as 40 % [11–13]. Thus there is a need for a non-invasive imaging modality that can achieve more accurate preoperative staging of bladder cancer.

Positron emission tomography/CT (PET/CT) is an approach in which the capacity to detect specific metabolic tissue changes is combined with simultaneous accurate anatomical depiction. Possible limitations of this combined technique are that the optimal isotope for studying bladder cancer has not yet been determined, and most isotopes have a short half-life and hence require use of an on-site cyclotron or a sophisticated transport modality [14, 15].

Most malignant tumors are characterized by elevated glucose metabolism [16, 17], and therefore increased cell proliferation in tumors can be imaged by PET/CT as increased uptake of 18-fluorodeoxyglucose (FDG). FDG is currently the only isotope approved by the US Food and Drug Administration for use in clinical oncology. FDG has a relatively short half-life (110 min), and it is excreted by the kidneys and accumulated in the urinary tract, which makes it difficult to study the urinary system in detail [18, 19]. Nevertheless, FDG is still the isotope that is most widely used to investigate bladder cancer [14].

In the present study, patients treated by radical cystectomy for locally advanced bladder cancer were investigated to compare the results of preoperative PET/CT and conventional CT with the findings of postoperative histopathological evaluation of LNs.

## Methods

### Patients

At our hospital, a total of 67 patients with urinary bladder cancer were scheduled for radical cystectomy with pelvic LN dissection between 2010 and 2012. All these patients underwent FDG-PET/CT and conventional CT of the thorax and abdomen as part of pre-cystectomy evaluation. Twelve of the patients did not have a cystectomy for the following reasons: 4 had distant metastasis (M1; positive in both conventional CT and PET/CT); 2 had ASA 4 and high surgical risk; 6 preferred radiation therapy. One of the remaining 55 patients did undergo radical cystectomy but had no LN dissection and was therefore excluded from further analysis. All patients were scheduled for FDG-PET/CT of the thorax and the abdomen 1 to 2 months before cystectomy. However, 6 patients had undergone PET/CT more than 2 months before cystectomy, because the surgery had been delayed to perform necessary investigations to address suspected distant metastases. No patient had preoperative chemotherapy.

The use of PET/CT as an additional mode of investigation of candidates for cystectomy was initiated at our department as a routine procedure in all patients. The method was considered to be safe and well established. We studied herein if additional information could be extracted from PET/CT in this particular setting. No ethical consent was therefore considered to be necessary.

However, we had prior to the start of the investigation an approval from The Regional Ethics Committee (Reference number M42-08). We used oral informed consent from the patients.

### Equipment and imaging protocol

The patients scheduled for FDG-PET/CT fasted at least 4 h before injection of the FDG. Blood glucose was monitored immediately before the injection, and a level lower than 8 mmol/L was required to perform the examination. The dose of FDG was 4 MBq/kg body weight. Images were acquired 60 min after the injection, and the patient drank 1 L of fluid or contrast medium during the 60-min interval between injection and imaging. The examination was done using a Siemens Biograph 40 PET/CT scanner with the patient in a dorsal recumbent position with the arms above the head. Full-dose CT with IV contrast was performed first, followed by PET (1.5–mm and 5–mm slices and a resolution of 4.2 mm). CT was performed using a thickness of 1.5 mm and a gap of 1.5 mm at 100 kV while mAS was regulated automatically according to the volume of the patient.

The majority of the patients (88 %) had an indwelling three-way catheter with bladder irrigation for continuous evacuation of the bladder. In patients without such a catheter, a renewed low-dose CT with PET was conducted approximately 30 min after the start of the initial imaging to detect PET-positive foci in the renal pelvis, the ureters, or the bladder after evacuation of all excreted FDG.

### Image interpretation

The PET/CT images were evaluated by two radiologists specialized and/or with extensive clinical experience in PET/CT using axial, coronal and sagittal reconstructions. These experts performed the assessments independently and without knowledge of operative or postoperative data. In cases in which the results obtained by the two radiologists were discordant, the investigations were mutually reviewed and discussed to reach consensus. Kappa analysis showed good agreement (Kappa value 0.85) between the PET/CT evaluations conducted by the two radiologists. Conventional CT and PET/CT were evaluated separately. The CT scans were assessed without knowledge of the PET findings. Positive LN findings were recorded for the following anatomical regions used in clinical practice for pelvic LN dissection: obturator fossa, external iliac, internal iliac, and common iliac. All PET images were evaluated by determining the maximum standardized uptake value (SUV), using a SUV-max cut-off of 2.5. LNs were considered to be positive by conventional CT if they had a diameter of ≥ 1 cm, and positive by PET/CT if they exhibited higher levels of activity than the SUV-max cut-off level regardless of their size. No pathological LNs were found in the pelvis outside the surgical templates. Distant metastases were suspected when higher levels of radioactivity were found in other organs, and in such cases evaluation was done to exclude metastasis before surgery.

### Surgery

Radical cystectomy was performed with a standardized LN dissection. The upper limit of the LN dissection was at the level of the ureteric crossing of the common iliac vein immediately cranial to confluence of the external and internal iliac veins in 49 (91 %) patients and was extended to the aortic bifurcation in 5 patients. The decision to extend the dissection was made by the surgeon if LN metastases were suspected based on the radiological, clinical, or perioperative data. The dissection extended down to the level of Cooper’s ligament, with the genitofemoral nerve as the lateral boundary. Specimens from the following four anatomical regions on both the right and the left side were sent separately for pathological examination: obturator, external iliac, and internal iliac, as well as the common iliac in cases with dissection to the aortic bifurcation. Each LN was sectioned in the middle along the larger axis, and a single 4-μm slice was mounted on a slide and stained with haematoxylin-eosin before analysis.

### Histopathological reevaluation

The specimens on the patients’ slides from cystectomy and lymph node dissection material were all reevaluated microscopically regarding T-stage, WHO grade, presence of lymphovascular invasion, and LN metastasis. This reassessment was performed by an experienced uropathologist (HO). All positive nodes were completely or almost completely infiltrated by bladder cancer.

### Data analysis

Results of PET/CT, conventional CT, and histopathological examination were compared on three levels: first, regarding positive or negative results in general for each patient regardless of node localization; second, according to the four anatomical regions; third, with respect to the side of the body (right or left) of each patient’s LN dissection. The third approach was applied because of potential difficulties associated with evaluating an exact anatomical region in both PET and surgery. Using the histopathological examination as gold standard, each PET/CT and conventional CT examination was classified as true positive or false positive, and true negative or false negative. The sensitivity, specificity, positive predictive value (PPV), and negative predictive value (NPV) were calculated.

## Results

Of the 54 evaluable patients, 50 (93 %) had urothelial cancer and 4 (7 %) had non-urothelial cancer. Also, 40 (74 %) of the 54 patients had muscle-invasive tumors. The mean time between PET/CT and surgery was 30 (6–133) days. Six patients had undergone PET/CT more than 2 months before cystectomy, because the surgery had been delayed for further investigations due to suspected distant metastasis. In one of these 6 patients, LNs were positive in histopathological examination but negative by both PET/CT and conventional CT; for the remaining 5 patients, all 3 investigations were negative. Thus 54 patients were eligible for evaluation in our study, 47 men and 7 women (mean age 68 years, range 46–85 years). Considering all 54, histopathological examination showed no LN metastasis (N0) in 37 (69 %) but revealed 1 or more positive LNs in 17 (31 %), and 16 (94 %) of those 17 patients had pT3-pT4 disease. FDG uptake was found in 12 patients (22 %), and 7 of those observations were confirmed by pathology. Conventional CT alone showed enlarged LNs in 11 patients (20 %), which was confirmed by pathology in 7 cases. Equivalent findings were obtained by both PET/CT and conventional CT in 43 (80 %) of the 54 patients (Table 1). The following was observed in the remaining 11 patients (20 %): 9 were positive by PET/CT and negative by conventional CT, and only 1 of those findings was confirmed by pathology; 2 were positive by conventional CT and negative by PET/CT, and neither case was confirmed by pathology.

**Table 1:** Patient characteristics

	Pathology findings		
	Positive	Negative	Total
	N	N	N
Gender			
Male	14	33	47
Female	3	4	7
Age			
Mean ≤ 68y	6	22	28
Mean > 68 y	11	15	26
Clinical stage			
cT1	1	8	9
cT2	6	21	10
cT3	10	7	16
cT4	0	1	14
Pathological stage			
pT1	0	14	14
pT2	1	9	10
pT3	7	9	16
pT4	9	5	14
PET/CT			
Positive	7	5	12
Negative	10	32	42
CT findings			
Positive	7	4	11
Negative	10	33	43

At the first level of analysis, which considered the whole patient, sensitivity was low for both PET/CT and conventional CT (Table 2). At the second level of analysis, we investigated all 1,518 LNs (mean 28 nodes per patient) excised from 347 sites in the stipulated anatomical regions, and metastases were confirmed by pathology in 99 LNs (7 % of all LNs) from 55 (16 %) of the 347 sites. FDG-PET/CT was negative in 41 sites with metastases, which was confirmed by pathology, whereas conventional CT was negative in 48 sites with metastases. Also at this level of analysis, sensitivity was low for both PET/CT and conventional CT (Table 2).

**Table 2:** Different levels of analysis of lymph nodes comparing pathology results and results of conventional CT and PET/CT in 54 patients treated with cystectomy for locally advanced bladder cancer

First level of analysis						
	Patho pos	Patho neg	Sensitivity	Specificity	PPV	NPV
CT pos	7	4	41 %	89 %	64 %	77 %
CT neg -	10	33				
PET pos+	7	5	41 %	86 %	58 %	76 %
PET neg-	10	32				
Second level of analysis						
	Patho pos	Patho neg	Sensitivity	Specificity	PPV	NPV
CT pos	7	10	13 %	97 %	41 %	85 %
CT neg	48	282				
PET pos	14	24	25 %	92 %	37 %	87 %
PET neg	41	268				
Third level of analysis						
	Patho pos	Patho neg	Sensitivity	Specificity	PPV	NPV
CT pos	9	5	31 %	94 %	64 %	79 %
CT neg	20	74				
PET pos	11	14	38 %	82 %	44 %	78 %
PET neg	18	65				

Thus there were difficulties in determining the exact location of individual LNs in precise anatomical regions, both during surgery and in radiological evaluation. Accordingly, in the third level of analysis, we considered both the left and the right side of in each patient, which showed low sensitivity for both PET/CT and conventional CT (Table 2).

## Discussion

In the present study, we compared FDG-PET/CT and conventional CT regarding the rate of detection of positive LNs as standard investigation of all patients before cystectomy, using histopathological examination of LNs as the gold standard. We found low levels of sensitivity for both FDG-PET/CT and conventional CT, which agrees with the results obtained by Swinnen et al. [20]. However, the levels we observed are lower than those reported by Drieskens et al, Liu et al, and Kibel et al, and Goodfellow et al [21–24], who noted sensitivity rates ranging from 60 to 77 % when using protocols for PET/CT imaging and analyses similar to those employed in our study. There is no obvious explanation for this discrepancy, although most of the mentioned studies were rather small, and hence only a few additional patients with positive PET/CT results might have had a marked impact on the rate of sensitivity.

Another plausible reason for differences in the LN detection rate might be related to the extent of LN dissection. In our series, we used the level of the ureteric crossing of the common iliac vein immediately cranial to confluence of the external and internal iliac veins as the upper limit of dissection in the majority of cases, whereas extended dissection to the aortic bifurcation was used in patients with clinical or radiological suspicion of LN metastases. A similar approach was applied by Kibel et al [22] with 70 % sensitivity. However, Swinnen et al [20] performed dissection to the aortic bifurcation in all patients and despite that observed sensitivity rates comparable to those noted in our series. These findings seem to suggest that the level of LN dissection does not determine the rate of sensitivity of PET/CT. Other investigators have discussed the completeness of LN dissection in terms of the number of extirpated LNs, using cut-offs of 16 and 20 LNs as an indication of a complete dissection [6, 11]. In our patients, a mean of 28 LNs were extirpated, which might represent an acceptable level of completeness in the dissection. Notably, studies of other comparable series have not given any information about the number of LNs removed [21–25].

Optimally, PET/CT should detect a localized pathological LN in order to enable regionally limited LN dissection [16, 20]. However, we found that sensitivity decreased from 41 % on a patient level to 25 % on a regional LN level, and Drieskens et al. [21] have also noted a drop in sensitivity from 60 % to 50 % on the regional level. Even though the indicated lower sensitivity implies that PET/CT is not suitable for exact localization of regional LN metastases, this hybrid imaging technique might nonetheless be useful to achieve improved tumor staging in individual patients, particularly if it is performed in combination with percutaneous biopsies [25]. Furthermore, PET/CT might aid detection of pathological LNs outside the surgical template. Although we did not find any pathological LN outside the surgical template in our small series this might be an advantage of PET/CT to limit LN dissection as suggested by others [16, 20].

Employing novel tracers might also enhance detection of LN metastases. Orevi et al obtained promising PET/CT results using 11C-choline to investigate 18 patients with bladder cancer [26], and Ahlström et al reported 11C-methionine to be superior to FDG in this context [27].

Moreover, 2 recent studies have shown that PET/MRI alone had a greater impact on clinical management compared to PET/CT alone [28, 29]. However we are still awaiting a systematic study PET/MRI in bladder cancer.

PET/CT has a number of limitations, and a potential drawback of our study concerns the difficulty in localizing the exact site of pathological LNs both at surgery and at PET/CT. The resolution of PET is inadequate and needs to be improved to provide better images, although tumor size was not a problem in our study as the image resolution was 4.2 mm and all our positive LNs were massively infiltrated with tumour cells [30]. Other potential weaknesses of our study include aspects related to tumor biology, such as the degree of glucose consumption, which varies considerably and is affected by multiple factors [16]. A major drawback of FDG-PET/CT is urinary elimination of FDG, which can be only partly neutralized by using a bladder washing/rinsing system in combination with diuretic medication and intravenous fluids [18]. It is possible that other isotopes (e.g. 11C-methionine or 11C-choline) can be more useful than FDG as the tracer in PET/CT investigation of bladder cancer [26].

The design of our study also had some limitations. This investigation comprising a small number of patients, and hence no firm conclusions can be drawn from the results. Furthermore, it was our intention to explore the possibility of routine PET/CT for all patients undergoing cystectomy. Considering the substantial number of PET/CT examinations that were negative in our subjects, it might have been more appropriate to study patients who were at greater risk of having positive LNs. Our assessments relied on pathological examination of single sections of LNs, and it is possible that using step-sectioned nodes for comparison would have improved the sensitivity and specificity of PET/CT. Finally, the time from PET/CT to surgery in our study was too long in some cases, which might have influenced the results.

## Conclusions

This study showed that, compared to conventional CT, FDG-PET/ CT provided no improvement in detection and localization of regional LN metastases in bladder cancer. Both these imaging approaches showed low sensitivity in detecting LN metastases, and the sensitivity decreased with a more exact degree of LN localization.
